# Long-term exposure to PGE_2_ causes homologous desensitization of receptor-mediated activation of protein kinase A

**DOI:** 10.1186/s12974-016-0645-0

**Published:** 2016-07-11

**Authors:** Ramy Habashy Malty, Andy Hudmon, Jill C. Fehrenbacher, Michael R. Vasko

**Affiliations:** Department of Chemistry and Biochemistry, Faculty of Science, University of Regina, Regina, SK Canada; Department of Biochemistry and Molecular Biology, Indiana University School of Medicine, Indianapolis, IN USA; Department of Pharmacology and Toxicology, Indiana University School of Medicine, Indianapolis, IN USA; Department of Pharmacology and Toxicology, Indiana University School of Medicine, 635 Barnhill Dr., A449, Indianapolis, IN 46202 USA

## Abstract

**Background:**

Acute exposure to prostaglandin E_2_ (PGE_2_) activates EP receptors in sensory neurons which triggers the cAMP-dependent protein kinase A (PKA) signaling cascade resulting in enhanced excitability of the neurons. With long-term exposure to PGE_2_, however, the activation of PKA does not appear to mediate persistent PGE_2_-induced sensitization. Consequently, we examined whether homologous desensitization of PGE_2_-mediated PKA activation occurs after long-term exposure of isolated sensory neurons to the eicosanoid.

**Methods:**

Sensory neuronal cultures were harvested from the dorsal root ganglia of adult male Sprague-Dawley rats. The cultures were pretreated with vehicle or PGE_2_ and used to examine signaling mechanisms mediating acute versus persistent sensitization by exposure to the eicosanoid using enhanced capsaicin-evoked release of immunoreactive calcitonin gene-related peptide (iCGRP) as an endpoint. Neuronal cultures chronically exposed to vehicle or PGE_2_ also were used to study the ability of the eicosanoid and other agonists to activate PKA and whether long-term exposure to the prostanoid alters expression of EP receptor subtypes.

**Results:**

Acute exposure to 1 μM PGE_2_ augments the capsaicin-evoked release of iCGRP, and this effect is blocked by the PKA inhibitor H-89. After 5 days of exposure to 1 μM PGE_2_, administration of the eicosanoid still augments evoked release of iCGRP, but the effect is not attenuated by inhibition of PKA or by inhibition of PI3 kinases. The sensitizing actions of PGE_2_ after acute and long-term exposure were attenuated by EP2, EP3, and EP4 receptor antagonists, but not by an EP1 antagonist. Exposing neuronal cultures to 1 μM PGE_2_ for 12 h to 5 days blocks the ability of PGE_2_ to activate PKA. The offset of the desensitization occurs within 24 h of removal of PGE_2_ from the cultures. Long-term exposure to PGE_2_ also results in desensitization of the ability of a selective EP4 receptor agonist, L902688 to activate PKA, but does not alter the ability of cholera toxin, forskolin, or a stable analog of prostacyclin to activate PKA.

**Conclusions:**

Long-term exposure to PGE_2_ results in homologous desensitization of EP4 receptor activation of PKA, but not to neuronal sensitization suggesting that activation of PKA does not mediate PGE_2_-induced sensitization after chronic exposure to the eicosanoid.

## Background

Prostaglandin E_2_ (PGE_2_) is a critical inflammatory mediator that contributes to acute and chronic pain by directly altering the sensitivity of sensory neurons to noxious and non-noxious stimuli [1, 2]. This eicosanoid is produced and released in the periphery by acute tissue injury, and its production is sustained during chronic inflammation [3–5]. Acute sensitization of sensory neurons by PGE_2_ occurs through activation of EP receptors that couple to the G_αs_/3′,5′-cyclic adenosine monophosphate (cAMP) signaling pathway [6]. Acute exposure to PGE_2_ increases the production of cAMP in sensory neurons [7, 8], and inhibition of protein kinase A (PKA) attenuates prostaglandin-induced hyperalgesia [9] and prostaglandin-induced increases in sodium currents [10, 11] and TRPV1 channel activity [12].

The signaling for chronic prostaglandin-mediated sensitization has been historically quite puzzling, since it is well established that chronic exposure to agonists can desensitize G-protein-coupled receptors (GPCRs) [13, 14]. However, an important characteristic of prostaglandin-induced hypersensitivity is that it does not downregulate with long-term exposure to the eicosanoid. For example, in patients with chronic inflammatory conditions, drugs that prevent the synthesis of prostaglandins (non-steroidal anti-inflammatory drugs, NSAIDs) are effective in reducing both acute and chronic hypersensitivity [15–17], suggesting that prostaglandins maintain their ability to sensitize sensory neurons through a mechanism that is not subject to classical GPCR downregulation. In animal models of inflammation or in animals chronically exposed to PGE_2_, the ability of the eicosanoid to enhance nociception does not diminish, but subsequent administration of PGE_2_ results in a stronger and more prolonged hyperalgesia [18–20]. This phenomenon, termed “hyperalgesic priming” [21], can be modeled in isolated sensory neurons where acute exposure to PGE_2_ sensitizes neurons to various stimuli [1, 7, 22] and, like their in vivo counterparts, the sensitizing actions of eicosanoids are not diminished by chronic exposure [23, 24].

Although the cellular mechanisms that account for persistent sensitization of sensory neurons by PGE_2_ are not known, one potential explanation for maintaining sensitization is through effector switching. For example, after an inflammatory insult, which increases production of prostaglandins and other inflammatory mediators, hyperalgesia induced by subsequent injection of PGE_2_ is not attenuated by inhibiting PKA but is blocked by inhibitors of other signaling effectors [20, 25]. After 14 daily injections of PGE_2_ into the rat hindpaw, hyperalgesia-induced by PGE_2_ injection is attenuated by PKA and protein kinase C_Ɛ_ inhibitors, not just by inhibiting PKA [18]. In sensory neurons from normal animals, the ability of PGE_2_ to augment ATP-induced current is blocked by PKA inhibitors, whereas in neurons from inflamed rats, the PGE_2_ effect is abolished only after inhibition of both PKA and protein kinase C (PKC) [26]. Furthermore, when isolated sensory neurons are maintained in culture with the inflammatory mediator, nerve growth factor (NGF), the ability of PGE_2_ to sensitize the neurons is not blocked by inhibition of PKA, whereas in neurons grown without NGF, PKA inhibition is effective [27]. These data suggest that PKA is not the major effector of persistent PGE_2_-induced sensitization of sensory neurons.

To date, there are few, if any, studies that directly examine whether chronic exposure to PGE_2_ downregulates the activation of PKA and, if so, whether this downregulation is specific for PGE_2_-induced activation. Consequently, using sensory neuronal cultures, we examined whether long-term exposure to PGE_2_ causes a loss in the ability of the eicosanoid to activate PKA. Our results show that chronic exposure of sensory neuronal cultures to PGE_2_ or an EP4 receptor agonist results in a complete but reversible loss in the ability of PGE_2_ to activate PKA. Furthermore, both acute sensitization and that which is observed after long-term exposure to PGE_2_ show the same profile of EP receptor activation suggesting that the downregulation is not secondary to alterations in EP receptor expression or function. This functional downregulation of PKA is homologous since activation of PKA by carbaprostacyclin, forskolin, or cholera toxin is not altered by chronic exposure to PGE_2_.

## Methods

### Materials

Fetal bovine serum, F-12 media, glutamine, penicillin-streptomycin, and fungizone were obtained from Invitrogen, Carlsbad, CA, whereas Normocin was purchased from InvivoGen, San Diego, CA. The small molecule PKA inhibitor H-89, the PKA pseudosubstrate inhibitor fragment 5-24 (PKI 5-24), Kemptide, poly-d-lysine, laminin, collagenase, 5-fluoro-2′-deoxyuridine, uridine, capsaicin, 1-methyl-2-pyrrolidinone (MPL), cholera toxin (CTX), TG4-155, and other routine chemicals were purchased from Sigma-Aldrich, St. Louis, MO. PGE_2_, carbaprostacyclin (cPGI_2_), L902688, ONO-8711, ONO-AE3-208, rabbit polyclonal antibodies for EP receptors, and cAMP enzyme immunoassay kits were purchased from Cayman Chemicals, Ann Arbor MI. L-798,106 was purchased from Santa Cruz, Dallas, TX. 3-isobutyl-1-methylxanthine (IBMX) and rat calcitonin gene-related peptide (CGRP) were obtained from Tocris Bioscience, Minneapolis, MN, and (Tyr27)-α-CGRP (27–37) was acquired from Bachem, Torrance, CA. [γP^32^]-ATP was purchased from PerkinElmer, Waltham, MA. Protease inhibitor cocktail Set III, EDTA-free, and phosphatase inhibitor cocktail set I were obtained from EMD Millipore, Darmstadt, Germany. LI-COR blocking buffer, TO-PRO-3, and Rockford secondary antibodies were obtained from LI-COR Biosciences, Lincoln, NE. Prestained protein size markers, precast SDS-PAGE gels, iScript reverse transcription kits, and PVDF membranes were obtained from BioRad, Hercules, CA. RNA STAT-60 was purchased from Tel-test, Inc., Friendswood, TX. Normal donkey serum was from Jackson ImmunoResearch Laboratories, West Grove, PA. NGF was purchased from Envigo, Indianapolis, IN. PGE_2_, cPGI_2_, L902688, forskolin, and capsaicin were initially dissolved in MPL and then diluted to the desired concentration with phosphate-buffered saline (PBS). Cholera toxin was dissolved in a buffer consisting of 0.05 M Tris buffer, pH 7.5, 0.2 M NaCl, 0.003 M NaN_3_, and 0.001 M sodium EDTA as per Sigma-Aldrich product information. Other drugs were diluted in PBS. The Animal Care and Use Committee at Indiana University School of Medicine, Indianapolis, IN, approved all procedures used in these studies.

### Cell culture

Sensory neuronal cultures were prepared as described previously with minor modifications [28]. Male Sprague-Dawley rats weighing approximately 145 g (Harlan, Indianapolis, IN) were euthanized by CO_2_ asphyxiation, and the dorsal root ganglia (DRG) were dissected from the entire spinal column and then incubated in F-12 media containing collagenase (1.25 mg/ml) for 1 hour at 37 °C. The collagenase-containing F-12 media was aspirated and replaced with fresh F-12 containing Normocin, and the DRG were mechanically dissociated using a fire-polished glass pipette. Cell culture wells were pre-coated with poly-d-lysine and laminin, and approximately 15,000 cells were plated into each well of 24-well culture plates, approximately 30,000 cells were plated into each well of 12-well culture plates, or approximately 60,000 cells were plated into each well of 6-well cultures plates. Cells were maintained in F-12 media supplemented with 10 % fetal bovine serum, 2 mM glutamine, 100 μg/ml Normocin, 50 μg/ml penicillin, 50 μg/ml streptomycin, 50 μM 5-fluoro-2′-deoxyuridine and 150 μM uridine in saturated humidity, and 3 % CO_2_ incubator at 37 °C. Cultures were grown in the absence or presence of 30 ng/ml exogenously added NGF, as indicated, and the media was changed every other day. For experiments involving long-term exposure to PGE_2_, media with fresh PGE_2_ was changed every 24 h.

### Neuropeptide release

For release experiments, neuronal cultures grown for 8–12 days were washed with HEPES buffer (25 mM HEPES, 135 mM NaCl, 3.5 mM KCl, 2.5 mM CaCl_2_, 1 mM MgCl_2_, 3.3 mM d-glucose, and 0.1 % bovine serum albumin, pH 7.4) at 37 °C. Cultures were incubated for 10 min in 0.4 ml HEPES buffer in the presence and absence of vehicle or drugs to determine resting release and then for 10 min in 0.4 ml of HEPES buffer containing 30 nM capsaicin in the presence or absence of vehicle or drugs to stimulate peptide release. A third incubation with HEPES buffer alone for 10 min was performed to confirm the return to resting release, which occurs in all experiments. At the end of the release protocol, the cells were hypotonically lysed by incubation for 10 min in 0.4 ml of 0.1 M HCl to extract total remaining CGRP in the culture. Release and content samples were aliquoted and assayed for immunoreactive CGRP (iCGRP) by radioimmunoassay as previously described [29]. Release data are presented as percent of total iCGRP content/10 min.

### Measurement of PKA activity

On the day of the experiment, the F-12 media in the cultures was replaced with drug-free fresh media and maintained for 20 min in the CO_2_ incubator. The cultures were then exposed to different drug treatments at 37 °C for 10 min, followed by two washes in ice-cold PBS. Cultures were lysed in 250 μl of ice-cold lysis buffer that contained β-glycerophsophate 25 mM, EGTA 1.25 mM, MgCl_2_ 10 mM, dithiothrietol 1 mM, ×2 protease inhibitors cocktail set III, NaCl 100 mM, and 1 % Triton-X 100. Cells were scraped, and the buffer was snap-frozen in liquid nitrogen, stored at −80 °C, and assayed within 24 h. After thawing, cell lysates were briefly sonicated followed by centrifugation at 16,100×*g* for 30 min at 4 °C. For each sample, 10 μl of the supernatant was added to 40 μl of the PKA activity assay buffer containing β-glycerophosphate 25 mM, EGTA 1.25 mM, MgCl_2_ 10 mM, NaCl 100 mM, dithiothrietol 0.5 mM, ×2 phosphatase inhibitor cocktail set I, ATP 100 μM, [γP^32^]-ATP (3 μCi/40 μl), and Kemptide 10 μM. The reaction was incubated at 30 °C for 5 min. At the end of the 5 min incubation, 20 μl of this reaction mixture was spotted on P81 filter paper discs (Whatman, GE Healthcare Life Sciences) and washed five times (5 min per wash) in 15 mM phosphoric acid. The bound radioactivity was measured using Cerenkov counting in a scintillation counter. PKA activity was measured as a function of incorporation of radioactive phosphate into Kemptide, a peptide that is selectively phosphorylated by PKA [30, 31]. Under these assay conditions, PKA-induced phosphorylation exhibits a linear relationship (*r*^2^ = 0.99) over time for up to 10 min (data not shown) indicating that the substrates, ATP and Kemptide, are not limiting during the 5 min of incubation used in our studies. PKA activity was measured in the presence or absence of the selective pseudosubstrate inhibitor, PKI 5-24 (5 μM) and the difference represented as selective PKA activity. The PKA data are calculated as the ratio of the treatment-activated PKA minus nonspecific activity (determined in the presence of PKI 5-24) divided by the maximum PKA activity (using 10 μM cAMP) minus its nonspecific activity (determined in the presence of cAMP and PKI 5-24).

### Measure of cAMP

Growth medium was aspirated from the culture dishes, and cells were washed twice with 0.4 ml of HEPES buffer containing 2 mM IBMX. After washing, cells were incubated in 0.4 ml HEPES buffer containing IBMX for 20 min in the absence or presence of vehicle, 1 μM PGE_2_, or 1 μM forskolin. The HEPES buffer was aspirated, and the cells were scraped into 300 μl 0.1 N HCl, boiled for 5 min, and centrifuged at 1200×*g* for 15 min. The supernatant was decanted, frozen, and lyophilized. Samples were resuspended, and immunoreactive cAMP was assayed using enzyme immunoassay kits from Cayman Chemical according to kit instructions. Data were expressed as pmol of cAMP per well.

### RNA isolation and quantitative real-time RT-PCR

To extract RNA, the growth medium was removed and RNA STAT-60 was added directly to the wells. The cell lysate was transferred to a RNase- and DNase-free 1.5 ml Eppendorf tube and allowed to sit for 5 min at room temperature before the addition of chloroform (0.2 ml/1 ml RNA STAT-60). The samples were vortexed briefly, stored at room temperature for 5 min, and centrifuged at 12,000×*g* for 15 min at 4 °C. The aqueous layer containing RNA was transferred to a fresh RNase- and DNase-free Eppendorf tube, and the RNA was precipitated overnight at room temperature by the addition of 0.5 ml isopropanol. The RNA precipitate was pelleted by centrifugation at 12,000×*g* for 15 min at 4 °C. The supernatant was removed, and the remaining RNA pellet was washed with 1 ml of 75 % ethanol. The mixture was centrifuged at 7500×*g* for 10 min at 4 °C, the ethanol removed, and the pellet allowed to dry until no moisture was evident in the tube. The RNA pellet was resuspended in 20 μl of water treated with diethyl pyrocarbonate (DEPC water), and a 1/20 dilution of the RNA was quantitated using a BioRad SmartSpec 3000.

Following RNA isolation, approximately 1.5 μg of RNA product, 2 units of DNase I, and reaction buffer (20 mM Tris-HCl, 2 mM MgCl_2_, 50 mM KCl) were incubated at room temperature for 15 min. The DNase was inactivated by incubation at 65 °C in the presence of 2.5 mM EDTA. Approximately 1.0 μg of total RNA was reverse transcribed using the iScript cDNA synthesis kit. The reaction mix included 15 μl of RNA (1.0 μg), 4 μl of iScript Reaction mix, and 1 μl of iScript Reverse Transcriptase. The reaction was incubated at 25 °C for 5 min, followed by 42 °C for 30 min, and 85 °C for 5 min. Reverse transcription products were diluted and real-time PCR performed on an ABI Prism 7700 Sequence Detector, using SYBR Green Amplitaq Master Mix (Thermo Fisher Scientific). The primers were designed to be selective for each of the PGE_2_ receptor subtypes and splice variants, and for GAPDH, which was used as an endogenous control. Primer sequences were as follows: EP1F: AACAGGCGGTAACGGCACAT, EP1R: CGCAGTCTGCCTGCAACCT (NM_013100; amplicon size 110 bp); EP3CF: TCGCTGAACCAGATCTTGGAT, EP3CR: CTGGAGACAGCGTTTGCTACC (D16443; amplicon size 91 bp); EP4F: CCCTCCTATACCTGCCAGACC, EP4R: CATGCGTACCTGGAAGCAAA (NM_032076; amplicon size 68 bp); and GAPDHF: TTCAATGGCACAGTCAAGGC, GAPDHR: TCCTGGAAGATGGTGATGGG (X02231; amplicon size 70 bp). Amplification was performed using universal PCR parameters. After completion of 40 cycles, the temperature was ramped from 60 to 95 °C over 20 min to establish a dissociation curve in each PCR experiment to verify that the fluorescence signal was due to a single amplicon amplification.

The relative standard curve method was used to quantitate relative changes in messenger RNA (mRNA) expression. Standard curves from 1- to 100-fold dilutions of the experimental control starting cDNA were prepared for both the genes of interest and for GAPDH. For each experimental sample (two replicates of two different dilutions), the amount of the gene of interest and GAPDH was determined by the appropriate standard curve. These concentrations were corrected for dilution and normalized to the amount of cDNA in the vehicle-treated control group.

### Li-Cor quantitative immunohistochemistry

Neuronal cultures grown in 24-well culture plates were treated as indicated. Immediately after treatment, the buffer containing drugs was aspirated and 4 % formalin in PBS was placed on the cells for 20 min. The fixed cells were then rinsed five times with PBS containing 0.5 % Triton X-100 for 5 min each rinse. Cells were treated with Triton X-100 and then blocked using a 1:1 dilution of the Li-Cor blocking buffer in PBS for at least 2 h. Primary antibodies to the EP1, EP3, and EP4 receptors were diluted in 50 % Li-Cor blocking buffer solution in PBS at 1:50–1:250. Cells were incubated in primary antibody overnight and then rinsed five times with PBS containing 0.5 % Tween-20. Some wells of cells were not incubated with primary antibody to determine the nonspecific actions of the secondary antibody, i.e., background staining. The secondary antibody, Rockford goat anti-rabbit antibody, conjugated to IRDye™ 800CW was diluted in the 1:1 Li-Cor blocking buffer solution in PBS at 1:800. TO-PRO-3, a nucleic acid stain that emits signal detected on the 700 channel of the infrared scanner, was added to the secondary antibody at a concentration of 1:2000. Cells were incubated in the secondary antibody and TO-PRO-3 for 2 h. This portion of the experiment was performed in the dark, as the infrared dyes can photobleach in a manner similar to fluorescent dyes. The secondary antibody was then removed, and the cells were washed five times with PBS containing 0.5 % Tween-20. The plates of cells were allowed to air-dry and were scanned for infrared signal.

The plates were scanned using the Odyssey Imager infrared scanner. The scan intensity was set at 5 for both the 700- and 800-nm channels, and the scan quality was set at a resolution of 169 μm for medium quality scans. Both the 700 channel and the 800 channel were scanned simultaneously. Background signal was subtracted from the wells that were incubated with primary antibody. The signal intensity at the 800 channel (EP signal) was normalized to the most intense EP well for each experimental group to control for differences in staining intensities between different plates. The percent of maximum EP intensity was then divided by the signal at the 700 channel (nucleic acid signal) to correct for possible differences in cell density. Data were expressed as percent of the maximal EP immunoreactivity: TO-PRO-3 immunoreactivity.

### Data analysis

Data are expressed as mean ± the standard error of the mean (SEM) for at least three independent experiments from separate harvests. Protein kinase A activity data were analyzed using one-way ANOVA followed by Bonferroni’s post hoc test or using Student’s *t* test as indicated. For cAMP content, mRNA, and protein expression, a paired Student *t* test was used to determine significant differences between control and treated wells. A *p* value of <0.05 was considered statistically significant in all experiments.

## Results

### Prostaglandin E_2_ and agents that increase production of cAMP augment PKA activity in sensory neuronal culture

**Fig. 1:**
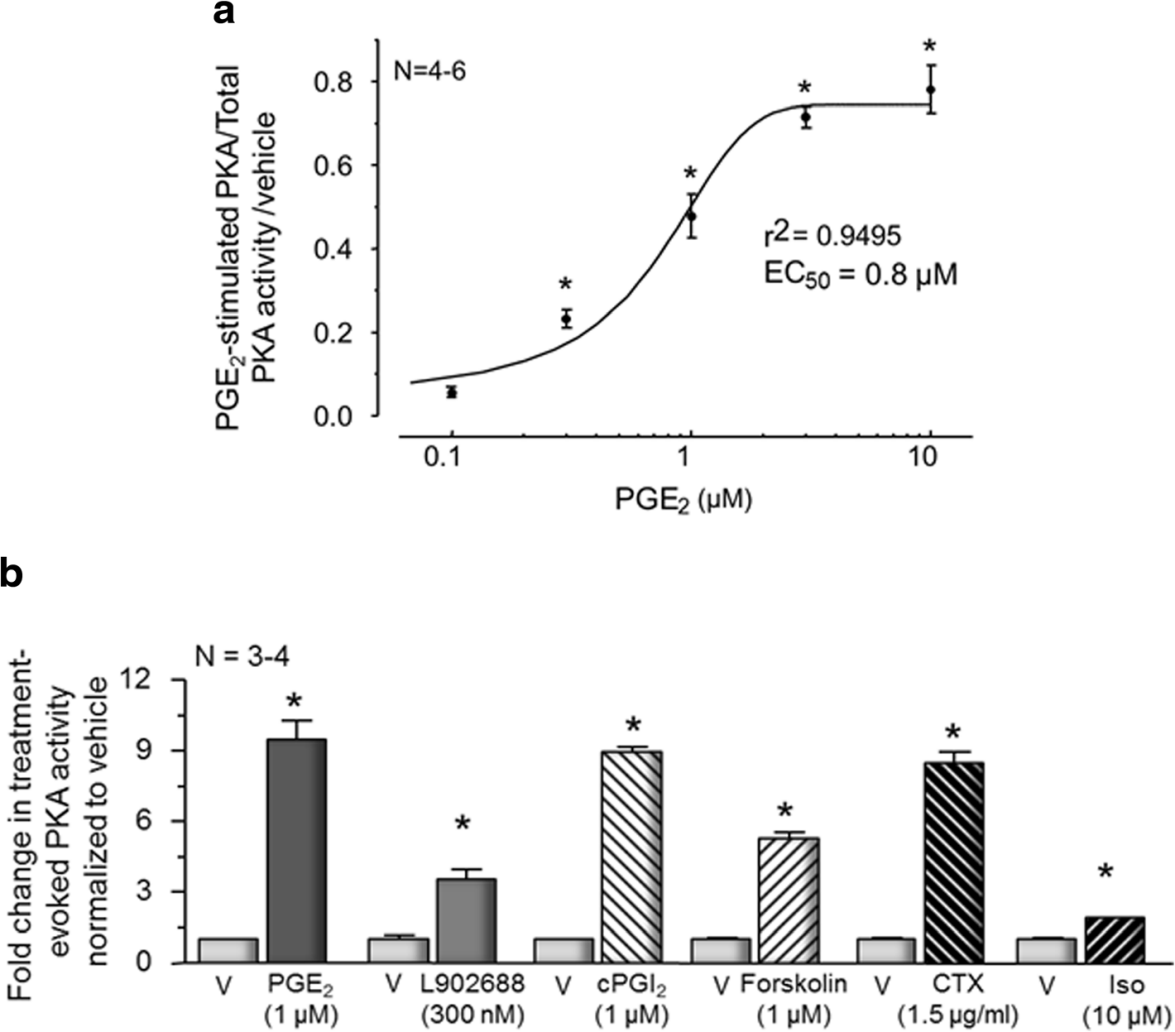
Prostaglandin E_2_ and other activators of cAMP production increase PKA activity in sensory neuronal cultures. **a** Each point represents mean ± SEM of PKA activity normalized to total PKA after 10-min exposure to various concentrations of PGE_2_ from 4 to 6 independent harvests of cells maintained in the absence of added NGF. *Asterisks* indicate a statistically significant increase in PKA activity compared to the vehicle-treated control using one-way ANOVA followed by Bonferroni’s post hoc test. **b** Each column represents the mean ± SEM of PKA activity normalized to total PKA after a 10-min exposure to vehicle (*V*), PGE_2_, the EP4 receptor agonist, L902688, cPGI_2_, forskolin, CTX, or isoproterenol (*Iso*) as indicated. An *asterisk* indicates a statistically significant difference between PKA activation by each treatment compared to its respective vehicle control using Student’s *t* test

Previous studies have shown that exposing sensory neurons in culture to PGE_2_ or prostaglandin I_2_ (PGI_2_) increases the production of cAMP [6, 7]. Furthermore, inhibitors of PKA attenuate the acute sensitizing actions of PGE_2_ suggesting that sensitization is mediated by activation of PKA [11, 12, 26, 32]. Because cAMP has multiple downstream effectors, we measured whether exposing sensory neuronal cultures to increasing concentrations of PGE_2_ would directly increase PKA activity (see the “Methods” section for details). When sensory neuronal cultures were exposed to PGE_2_ for 10 min and PKA activity determined in cell lysates, treatment with PGE_2_ resulted in a concentration-dependent increase in PKA activity (Fig. 1a). The relationship between the log concentration of PGE_2_ and PGE_2_-induced PKA activity was fit to a sigmoidal curve with a correlation coefficient of 0.95 and an EC_50_ of 0.8 μM. The normalized PKA activity increased from 0.06 ± 0.01 for cultures treated with 0.1 μM PGE_2_ to 0.78 ± 0.10 for cultures exposed to 10 μM PGE_2_. Concentrations of 0.3, 1, 3, and 10 μM PGE_2_ all produced a significant increase in PKA activity compared to vehicle (Fig. 1a).

Previously, we showed that the acute sensitizing actions of PGE_2_ on sensory neurons are mediated, in part, by activation of the EP4 receptors, which are coupled to G_αs_ [6]. Furthermore, increasing cAMP production via exposure of sensory neurons to cPGI_2_, which increases activation of G_αs_ through the IP receptor; forskolin, which is a direct activator of adenylyl cyclase; or CTX, which ADP-ribosylates G_αs_, also sensitizes sensory neurons [7]. Consequently, we examined whether these various drug treatments enhance PKA activity in our neuronal cultures. Exposing the cultures to 1 μM PGE_2_ increases PKA activity ~ninefold above that seen in vehicle-treated cells (0.01 % MPL; Fig. 1b), whereas 300 nM of the EP4 receptor agonist, L902688, increased PKA activity ~3.5-fold and 1 μM cPGI_2_ increased activity ~ninefold (Fig. 1b). Activation of adenylyl cyclases with 1 μM forskolin or exposure of cultures overnight to 1 μM CTX to activate G_αs_ also significantly increased PKA activity ~five- and ~ninefold compared to vehicle, respectively (Fig. 1b). We also examined whether activation of β-adrenergic receptors with isoproterenol would increase PKA activity since this drug when injected into the hindpaw of rats augments nociceptive behaviors [33]. Exposing neuronal cultures to 10 μM isoproterenol produced a small increase in PKA (1.2-fold) above vehicle-treated cultures (Fig. 1b). Although significant, only modest PKA activation was observed following exposure to a range of isoproterenol concentrations (1–100 μM). The ratios of isoproterenol-activated PKA to total PKA activity were 0.12 ± 0.01, 0.10 ± 0.004, 0.11 ± 0.01, 0.13 ± 0.01, and 0.11 ± 0.003 for 1, 3, 10, 30, and 100 μM, respectively (data not shown).

### PGE_2_-induced augmentation of capsaicin-evoked iCGRP release is maintained after long-term exposure to the eicosanoid but is not mediated by activation of PKA

**Fig. 2:**
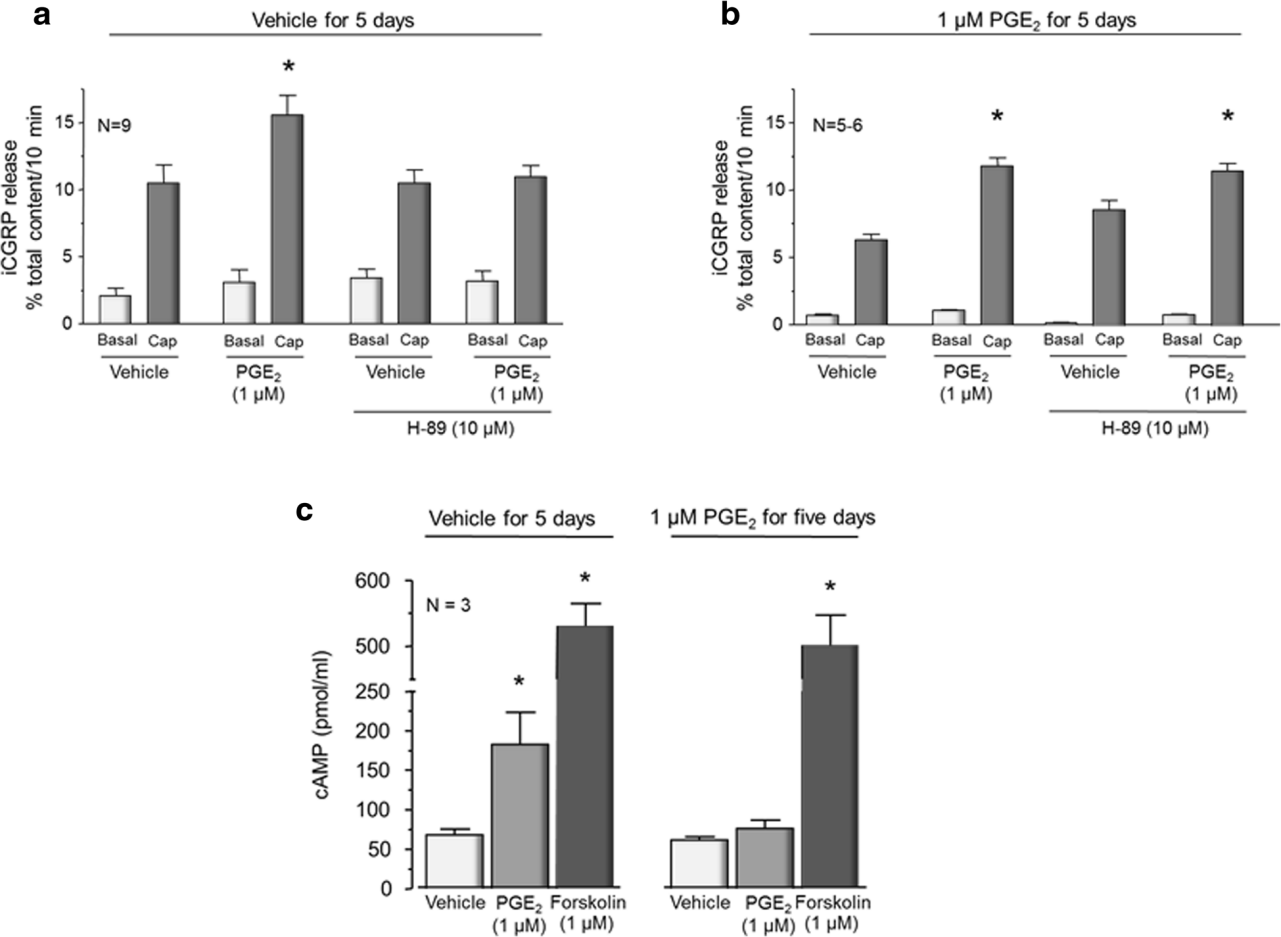
The PGE_2_-induced increase of capsaicin-evoked iCGRP release from sensory neurons is attenuated by H-89 after acute exposure to the eicosanoid but not after long-term exposure. **a**, **b** Each column represents the mean ± SEM of iCGRP release as percent of total iCGRP content per well of cells maintained in the absence of added NGF. *Lightly shaded columns* indicate basal release whereas *dark-shaded columns* represent capsaicin-stimulated release of iCGRP. Cultures were treated with vehicle (**a**) or 1 μM PGE_2_ (**b**) for 5 days and then washed and acutely exposed to 1 μM PGE_2_ for 20 min in the absence or presence of 10 μM H-89, as indicated. An *asterisk* indicates a statistically significant difference between capsaicin-stimulated iCGRP release after exposure to vehicle versus after a 10-min exposure to PGE_2_ (1 μM) using one-way ANOVA followed by Bonferroni’s post hoc test. **c** Each column represents the mean ± SEM of cAMP content. The *left panel* represents cAMP content from cells exposed to vehicle for 5 days, whereas the *right panel* represents cAMP content from cells exposed to PGE_2_ (1 μM) for 5 days. Cultures were washed and then re-exposed for 10 min to vehicle (*open columns*), 1 μM PGE_2_ (*light gray columns*), or 1 μM forskolin (*dark gray columns*). An *asterisk* indicates a statistically significant difference from vehicle using one-way ANOVA followed by Bonferroni’s post hoc test

Since the acute sensitizing action of PGE_2_ on sensory neurons is mediated through activation of EP receptors that are part of the GPCR family [6, 34], chronic exposure to PGE_2_ should result in tolerance or desensitization to the sensitizing effects of this prostanoid. Previous studies, however, suggest that the ability of PGE_2_ to sensitize sensory neurons does not downregulate after chronic exposure to the eicosanoid [17, 24]. Consequently, we examined whether the ability of PGE_2_ to augment capsaicin-evoked release of iCGRP from sensory neurons downregulated after long-term exposure to the prostanoid and whether this sensitizing action was attenuated by the PKA inhibitor, H-89. When sensory neurons in culture were exposed to 30 nM capsaicin, release of iCGRP increased approximately threefold from a basal level of 3.4 ± 0.7 % of total content/10 min to 10.5 ± 1.3 % of total content/10 min (Fig. 2a). Treating cells with 1 μM PGE_2_ significantly augmented the capsaicin-evoked release to 15.6 ± 1.4 % of total content/10 min (Fig. 2a). Although exposure to 10 μM H-89 did not alter basal or capsaicin-stimulated release of iCGRP, it did block the ability of PGE_2_ to augment capsaicin-evoked release (Fig. 2a).

To examine the effects of long-term exposure to PGE_2_, we treated sensory neuronal cultures with 1 μM PGE_2_ for 5 days. For these studies, we replaced the culture media with media containing fresh PGE_2_ every 24 h since previous studies demonstrated that PGE_2_ levels are maintained after 24 h in culture [35]. When neuronal cultures were treated with 1 μM PGE_2_ for 5 days prior to examining iCGRP release and the cells re-exposed to 1 μM PGE_2_ for 20 min, the eicosanoid significantly increased the capsaicin-evoked release from a control level of 6.2 ± 0.4 to 11.6 ± 0.6 % of total content/10 min (Fig. 2b) demonstrating that long-term exposure to PGE_2_ does not downregulate the sensitizing actions of the prostanoid. Exposing sensory neuronal cultures to 1 μM PGE_2_ for 5 days did not alter the total content of iCGRP. Total peptide content in neuronal cultures exposed to vehicle for 5 days was 486 ± 58 fmol/well, whereas in cultures exposed to PGE_2_ for 5 days content was 540 ± 67 fmol/well. Thus, using enhancement of iCGRP release as an endpoint of neuronal sensitization, long-term exposure to PGE_2_ did not downregulate the sensitizing actions of the prostanoid. Although H-89 prevented the acute sensitizing effects of PGE_2_, PGE_2_-induced sensitization after long-term exposure to PGE_2_ was not blocked by pretreating the cultures with 10 μM H-89 (Fig. 2b). In the presence of 10 μM H-89 alone, capsaicin-evoked release of iCGRP was 8.5 ± 0.7 % of total content/10 min, whereas release from cells treated with 10 μM H-89 and 1 μM PGE_2_ was 11.3 ± 0.5 % of total content/10 min. These data support the notion that sensitization of sensory neurons by PGE_2_ after chronic exposure to the prostanoid is not dependent on the activation of PKA.

Since PGE_2_-induced sensitization is maintained after long-term exposure to the drug (Fig. 2b), and since acute exposure to the eicosanoid increases cAMP production [7], we measured cAMP levels directly to address the question of whether exposing neuronal cultures to 1 μM PGE_2_ for 5 days would alter the ability of the prostanoid to augment the production of cAMP. In neuronal cultures exposed to vehicle for 5 days, a 10-min treatment with 1 μM PGE_2_ significantly increased the content of cAMP from 68 ± 7 to 183 ± 40 pmol/ml (Fig. 2c). In cultures exposed to 1 μM PGE_2_ for 5 days, the content of cAMP after acute treatment with vehicle was 61 ± 4 pmol/ml and the cAMP content in cells re-exposed to PGE_2_ was 76 ± 10 pmol/ml. These values were not significantly different from cAMP content in cells treated with vehicle for 5 days. In contrast, the ability of forskolin to increase cAMP content was not significantly different in cultures exposed for 5 days to vehicle (530 ± 34 pmol/ml) or to 1 μM PGE_2_ (501 ± 46 pmol/ml).

### Acute PGE_2_-induced sensitization and persistent sensitization after long-term exposure to the eicosanoid are mediated by the same EP receptor subtypes

**Fig. 3:**
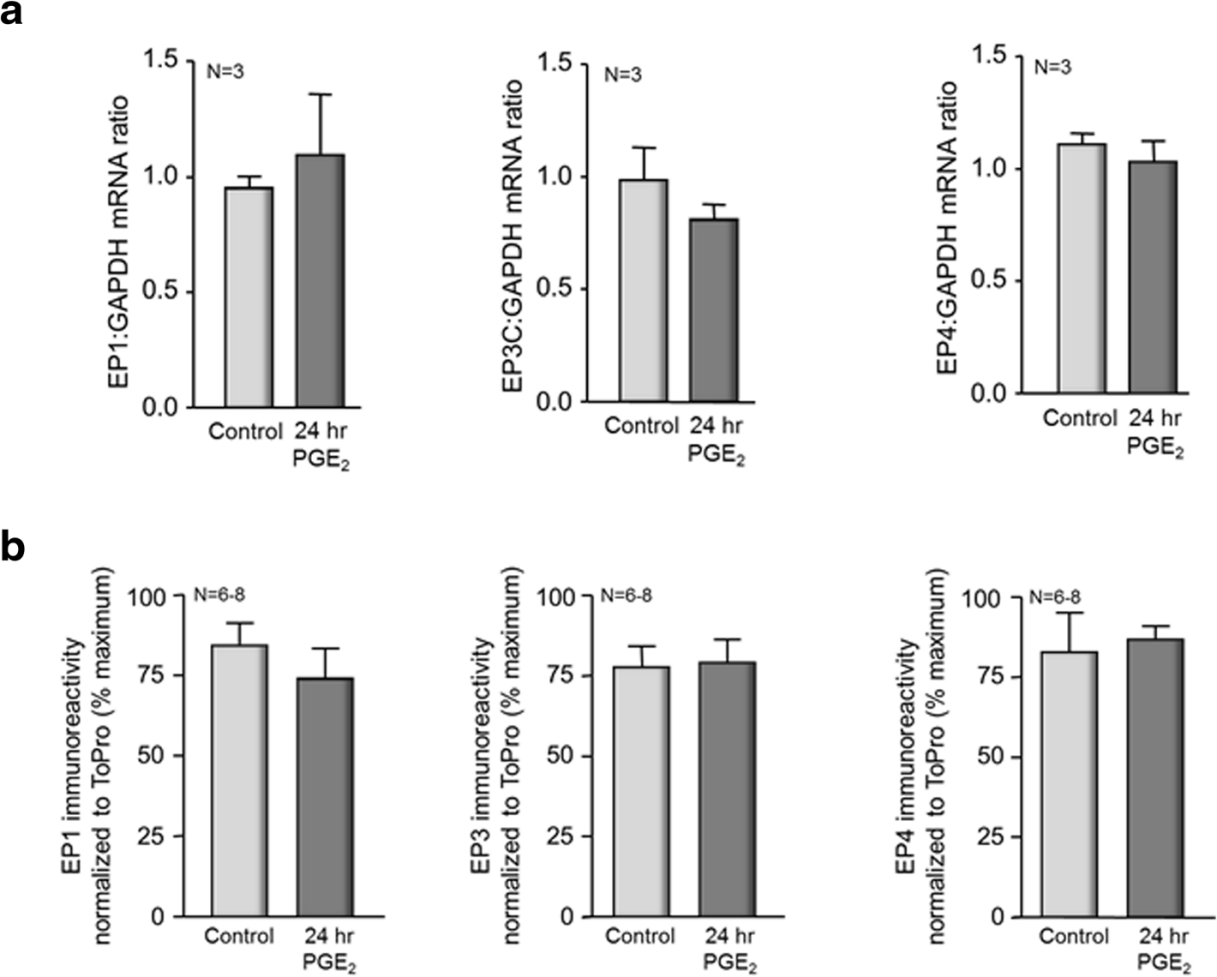
Twenty-four hour exposure of sensory neurons in culture to 1 μM PGE_2_ does not alter the expression of EP receptors as measured by real-time RT-PCR. **a** Dorsal root ganglia were harvested and cultured for 6 days in media containing 30 ng/ml NGF. After 24-h treatment with vehicle (*lightly shaded columns*) or 1 μM PGE_2_ (*dark-shaded columns*), the RNA was isolated and reverse transcribed to cDNA for analysis by PCR. Each column represents the mean ± SEM of the ratio of EP receptor mRNA to GAPDH mRNA from three independent harvests of cells. **b** Each column represents the mean ± SEM of the % maximum immunoreactivity for EP receptors normalized to TO-PRO-3 immunoreactivity in neuronal cultures after 24 h treatment with vehicle (*lightly shaded columns*) or 1 μM PGE_2_ (*dark-shaded columns*) for six to eight wells of cells

The data presented above suggest that the maintenance of PGE_2_ sensitization following chronic exposure to the prostanoid may be mediated by alternate EP receptors which couple to different G-proteins and activate alternate downstream signaling pathways. To examine this possibility, we measured mRNA for the EP1, EP3C, and EP4 receptors. We chose to study these receptor subtypes since our previous work suggests that EP3C and EP4 receptors contribute to acute sensitization in isolated sensory neurons [6]. Furthermore, acute sensitization by PGE_2_ has been proposed to be mediated through activation of EP1 receptors [36]. Six days after harvesting, sensory neuronal cultures were exposed to 1 μM PGE_2_ or vehicle for 24 h, and then total RNA was isolated from the treated cells and reverse transcribed to cDNA. Exposing cultures to PGE_2_ for 24 h did not significantly alter the amounts of mRNA for any of the EP receptors examined: EP1, EP3C, and EP4 (Fig. 3a). The levels of mRNA for the EP1 receptor normalized to mRNA for GAPDH were 0.95 ± 0.05 in control cells and 1.10 ± 0.26 after a 24-h treatment with PGE_2._ Levels of mRNA for EP3C and EP4 were 0.99 ± 0.14 and 1.11 ± 0.05 in control cells and 0.81 ± 0.07 and 1.03 ± 0.09, respectively, in cells treated with PGE_2_. Similar results were observed from neuronal cultures exposed to PGE_2_ for 5 days. In these cultures as in cultures treated for 24 h, long-term exposure to 1 μM PGE_2_ did not significantly alter mRNA to EP1, EP2, EP3, or EP4 receptors compared to cells treated with vehicle for 5 days (data not shown).

We also determined whether a 24 h exposure to PGE_2_ would alter the expression of EP receptor proteins using quantitative immunohistochemistry (see the “Methods” section). Analogous to the observations of mRNA expression, long-term exposure of sensory neurons to PGE_2_ did not alter EP receptor protein levels (Fig. 3b). The EP1 immunoreactivity in control wells was 84 ± 7 %, whereas immunoreactivity was 74 ± 9 % of the maximal signal after treatment. A 24 h exposure of sensory neurons to PGE_2_ did not alter total EP_3_ immunoreactivity; the control value of EP3 immunoreactivity was 78 ± 7 %, whereas the EP3 immunoreactivity value was 79 ± 7 % of maximal after a 24 h exposure to PGE_2_. Similarly, there was no change in EP4 immunoreactivity. Intensity values for EP4 protein were 83 ± 12 and 87 ± 4 % of maximal in the absence and presence of long-term treatment with PGE_2_, respectively (Fig. 3b). Together, the real-time PCR and quantitative immunohistochemistry data suggest that a 24 h exposure of sensory neurons in culture to PGE_2_ does not alter the expression of EP receptors.

**Fig. 4:**
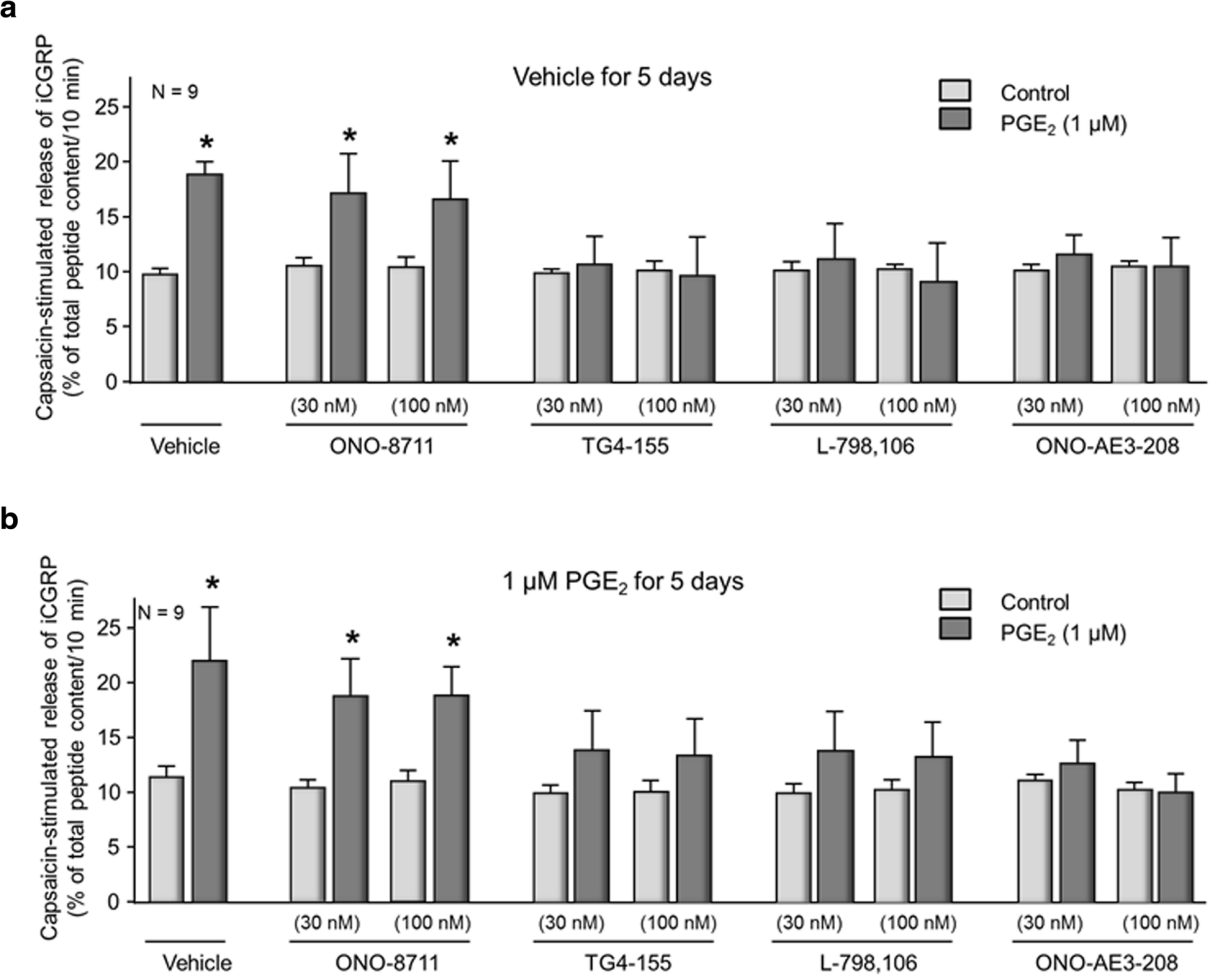
Acute PGE_2_-induced sensitization and persistent sensitization after long-term exposure to the eicosanoid are mediated by the same EP receptor subtypes. Each column represents the mean ± SEM of capsaicin-stimulated release of iCGRP as percent of total iCGRP content per well/10 min in cultures maintained in the absence of added NGF and preexposed to vehicle (**a**) or to 1 μM PGE_2_ for 5 days (**b**). After pretreatment, cultures were acutely exposed to vehicle (*lightly shaded columns*) or to 1 μM PGE_2_ (*dark-shaded columns*) for 20 min in the absence or presence of EP receptor antagonists, as indicated. An *asterisk* indicates a statistically significant difference between capsaicin-stimulated release of iCGRP after vehicle versus after PGE_2_ using one-way ANOVA followed by Bonferroni’s post hoc test

To identify the EP receptor subtypes that contribute to PGE_2_-induced sensitization, we used the selective EP receptor inhibitors ONO-8711, TG4-155, L798,106, and ONO-AE3-208 to block EP1, EP2, EP3, or EP4 receptor subtypes, respectively. In sensory neuronal cultures that were exposed to vehicle for 5 days, pretreating with 30 or 100 nM of the EP2 receptor antagonist, TG4-155; the EP3 receptor antagonist, L798,106; or the EP4 receptor antagonist, ONO-AE3-208, blocked the PGE_2_-induced augmentation of capsaicin-stimulated release of iCGRP (Fig. 4a). Exposure to the antagonists in the absence of PGE_2_ did not alter basal or capsaicin-stimulated release of iCGRP (Fig. 4a). In contrast, pretreating cultures with the EP1 receptor antagonist, ONO-8711, did not attenuate the PGE_2_-induced increase in capsaicin-stimulated release (Fig. 4a). After 5-day exposure to PGE_2_, re-exposure to the prostanoid caused sensitization that was completely inhibited by the EP4 receptor antagonist and to a lesser degree by EP2 and EP3 receptor antagonists, but not by the EP1 receptor antagonist (Fig. 4b). Together, these results show that chronic exposure to PGE_2_ does not change the EP receptor profile that mediates sensitization by the eicosanoid.

### Long-term exposure to PGE_2_ downregulates PKA activity induced by the prostanoid

**Fig. 5:**
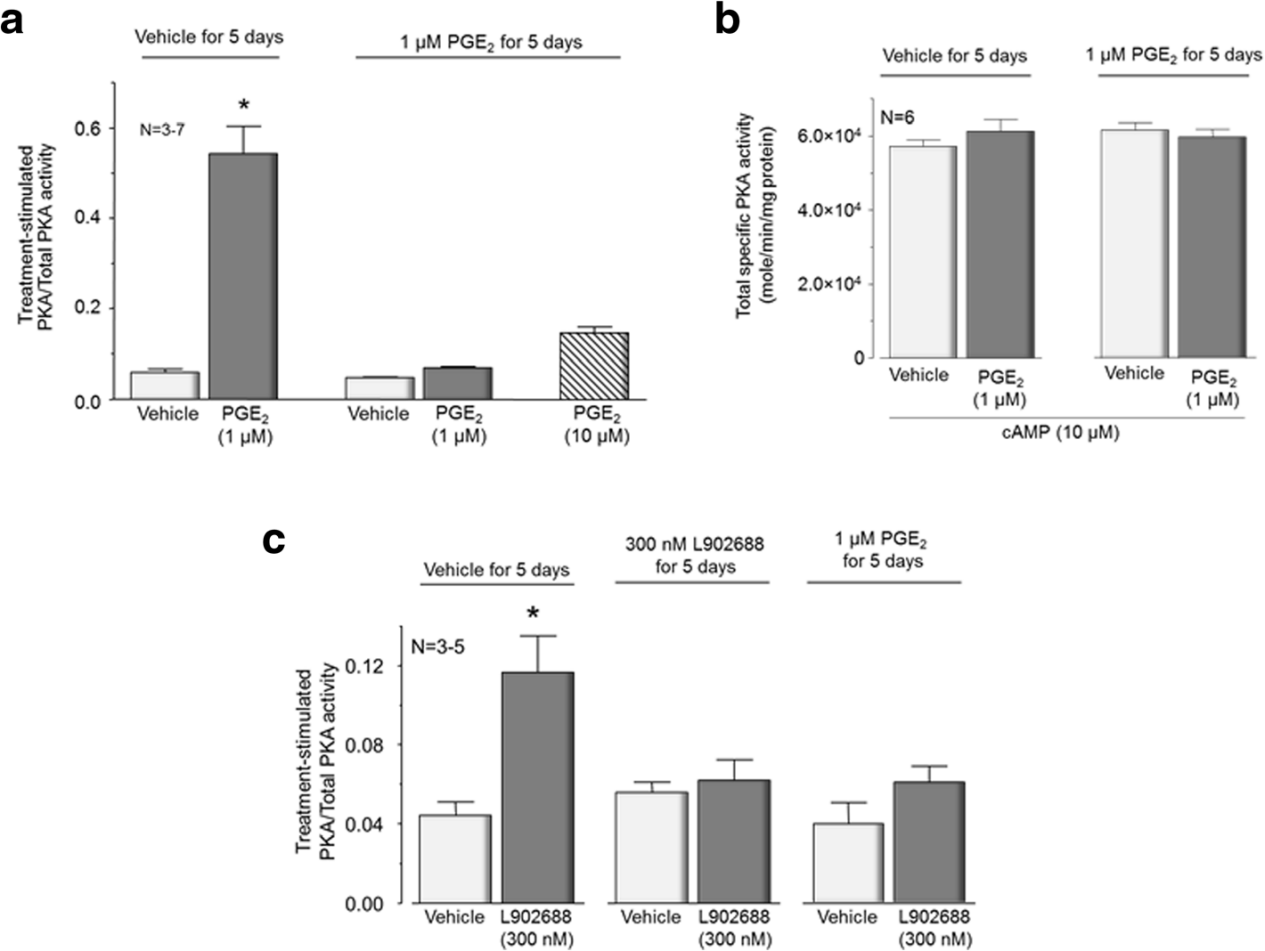
Exposing sensory neuronal cultures to PGE_2_ or to the EP4 receptor agonist L902688 for 5 days desensitizes the agonist-induced increase in PKA activity. **a** Each column represents the mean ± SEM of the treatment-stimulated PKA activity normalized to total PKA activity from cultures grown in the absence of added NGF and treated with vehicle or PGE_2_ (1 μM) for 5 days as indicated. Cultures were washed and then re-exposed for 10 min to vehicle (*open columns*), 1 μM PGE_2_ (*closed columns*) or 10 μM PGE_2_ (*hatched column*). **b** Each column represents mean ± SEM of total specific activity of PKA after exposure to 10 μM cAMP from cultures treated with vehicle or PGE_2_ (1 μM) for 5 days as indicated. Cultures were washed then re-exposed for 10 min to vehicle (*open columns*) or 1 μM PGE_2_ (*closed columns*). **c** Each column represents the mean ± SEM of the treatment-stimulated PKA activity normalized to total PKA activity from cultures treated with vehicle, 300 nM L902688, or PGE_2_ (1 μM) for 5 days as indicated. Cultures were washed and then re-exposed for 10 min to vehicle (*open columns*) or 300 nM L902688 (*closed columns*). An *asterisk* indicates a statistically significant difference from vehicle using one-way ANOVA followed by Bonferroni’s post hoc test

Although PGE_2_-induced sensitization of sensory neurons after long-term exposure to the eicosanoid does not appear to be PKA dependent, the question remains whether the ability of PGE_2_ to increase PKA activity downregulates with chronic exposure to the prostanoid. To examine this directly, we determined whether 1 μM PGE_2_ could increase PKA activity in neuronal cultures treated with the eicosanoid for 5 days. As observed in previous experiments, when sensory neuronal cultures were exposed to vehicle for 5 days and then challenged with 1 μM PGE_2_ for 10 min, the eicosanoid caused a significant increase in PKA activity from 0.06 ± 0.003 to 0.52 ± 0.1 (Fig. 5a). In contrast, when cultures are exposed to 1 μM PGE_2_ for 5 days and then re-exposed to the eicosanoid, there was no significant increase in PKA activity (PKA activity was 0.07 ± 0.0003, Fig. 5a). Increasing the concentration of PGE_2_ 10-fold caused a small, but not significant, increase in PKA activity (0.14 ± 0.01) in cultures exposed to PGE_2_ for 5 days (Fig. 5a). The total specific PKA activity after exposure to 10 μM cAMP was not affected by the long-term exposure to PGE_2_ suggesting that the downregulation of PGE_2_-activated PKA was not caused by any decrease in the overall kinase activity (Fig. 5b).

Since activation of EP4 receptors on sensory neurons mediates the sensitizing actions of PGE_2_ [6, 34], we examined whether long-term exposure to the EP4 receptor agonist L902688 or to PGE_2_ downregulated the increase in PKA activity produced by activation of EP4 receptors. We chose to use L902688 in the current experiments because it has an approximate 7000–32,000 higher affinity of binding to EP4 when compared to other EP receptor subtypes [37]. When neuronal cultures were exposed to 300 nM L902688 for 10 min, there was a significant increase in PKA activity from 0.04 ± 0.007 to 0.12 ± 0.01 (Fig. 5c). In contrast, when neuronal cultures were treated with L902688 for 5 days, re-exposure to the agonist did not significantly increase PKA activity above control levels (Fig. 5c). Likewise, when neuronal cultures were treated with 1 μM PGE_2_ for 5 days, exposing the cultures to L902688 for 10 min did not increase PKA activity (0.04 ± 0.01 and 0.06 ± 0.01 vehicle and L902688, respectively). Together, these data suggest that chronic activation of EP4 receptors results in a loss of their ability to couple to PKA signaling in response to an agonist.

### Time course of the onset and offset of desensitization of the PGE_2_-induced activation of PKA in sensory neuronal cultures after chronic exposure

**Fig. 6:**
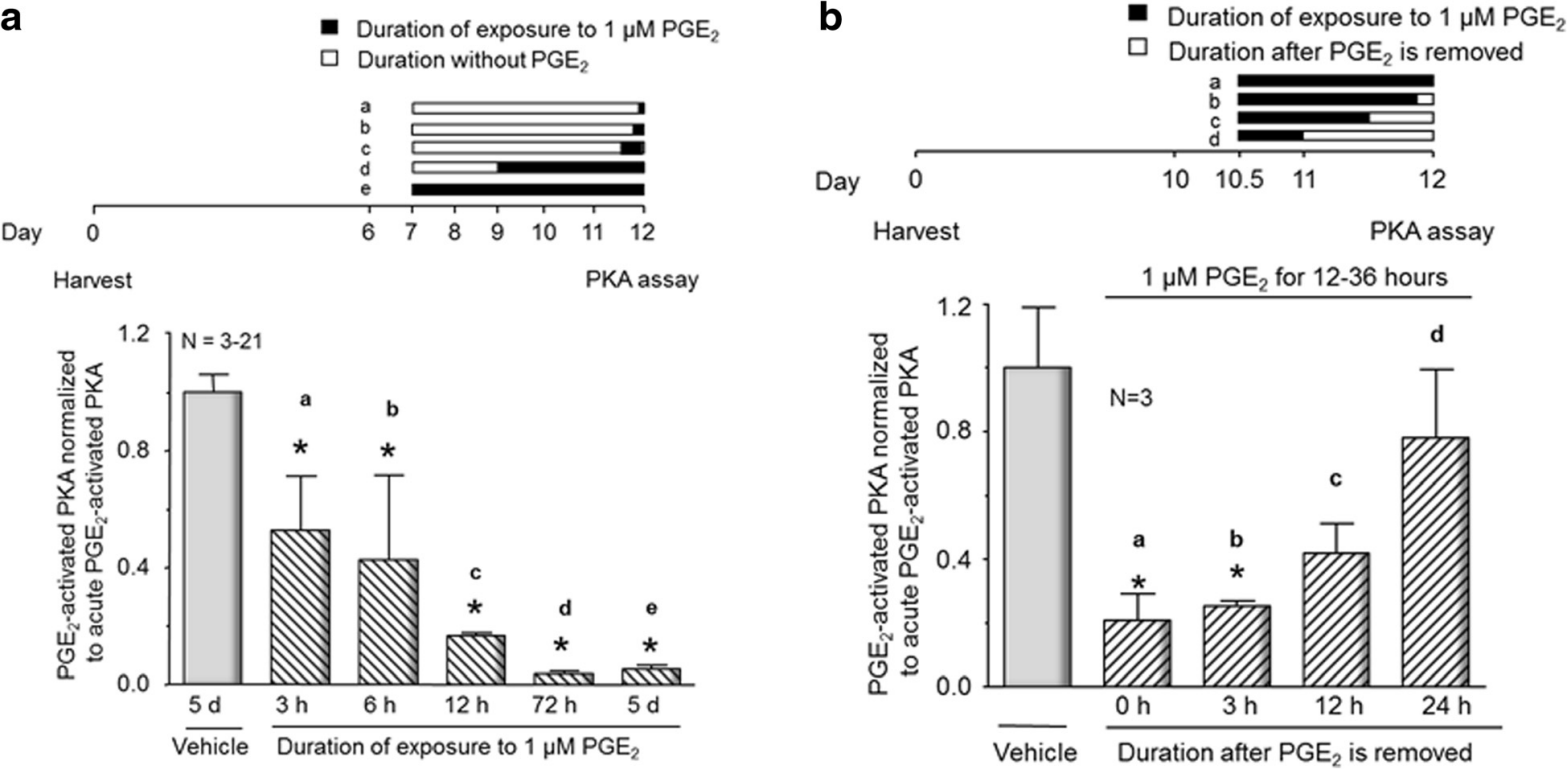
Time course of the onset and offset of desensitization of the PGE_2_-induced activation of PKA in sensory neuronal cultures after chronic exposure to the eicosanoid. Each column represents the mean ± SEM of PKA activity normalized to total PKA activity for various times of exposure to 1 μM PGE_2_ or after removal of 1 μM PGE_2_. **a** Cultures were pretreated with vehicle (*open column*) or 1 μM PGE_2_ (*hatched columns*) for the times indicated in the time line in the top portion of the figure. **b** Cultures were pretreated with vehicle (*open column*) or PGE_2_ (*hatched columns*) for the times indicated in the time line in the top portion of the figure, and then, the PGE_2_ is removed and cells assayed at the times indicated. An *asterisk* indicates a statistically significant difference between PGE_2_-treated sensory neuronal cultures and vehicle-treated cultures using one-way ANOVA followed by Bonferroni’s post hoc test

The data presented above clearly show that exposing sensory neurons to PGE_2_ for 5 days abolishes the subsequent PGE_2_-induced activation of PKA. To ascertain the duration of exposure to PGE_2_ that is necessary to downregulate prostanoid-induced activation of PKA and to determine whether this desensitization is reversible, we examined PGE_2_-induced PKA activation after cultures were exposed to PGE_2_ for various lengths of time. To determine the time course for desensitization, sensory neuronal cultures were exposed to either vehicle for the last 5 days in culture or to 1 μM PGE_2_ for the last 3, 6, 12, 72 h or 5 days in culture (Fig. 6a, top panel). In all instances, PKA activity was determined after cells were maintained in culture for 12 days. Three and 6 h exposures of neuronal cultures to PGE_2_ resulted in a reduction in the ability of PGE_2_ to activate PKA by approximately 50 % (Fig. 6a). After a 12 h exposure, the PGE_2_-induced PKA activity is reduced by 80 %, whereas maximal inhibition is observed after 3 days of exposure (Fig. 6a). To examine whether the desensitization was reversible, sensory neurons in culture were exposed to vehicle for 36 h or to 1 μM PGE_2_ for 36, 33, 24, or 12 h and then to vehicle for 0, 3, 12, or 24 h, respectively (Fig. 6b, top panel), and PKA activity was measured. All cells were maintained in culture for 12 days. Exposure of sensory neurons to 1 μM PGE_2_ for 36 h resulted in desensitization of PGE_2_-induced activation of PKA (Fig. 6b), an effect we also observed after 72 h and 5 days of treatment with the eicosanoid (Fig. 6a). Three hours after the PGE_2_ is removed, a re-exposure to the eicosanoid did not augment PKA activity (Fig. 6b). In contrast, 12 and 24 h after removal of PGE_2_, PKA activation by re-exposure to the eicosanoid recovered to approximately 42 and 78 % of PGE_2_-activated PKA in naïve cultures (Fig. 6b). Thus, downregulation of the PGE_2_-induced activation of PKA is reversible and not secondary to loss of cell viability after chronic exposure to PGE_2_.

### Homologous desensitization of PKA signaling after long-term exposure to PGE_2_

**Fig. 7:**
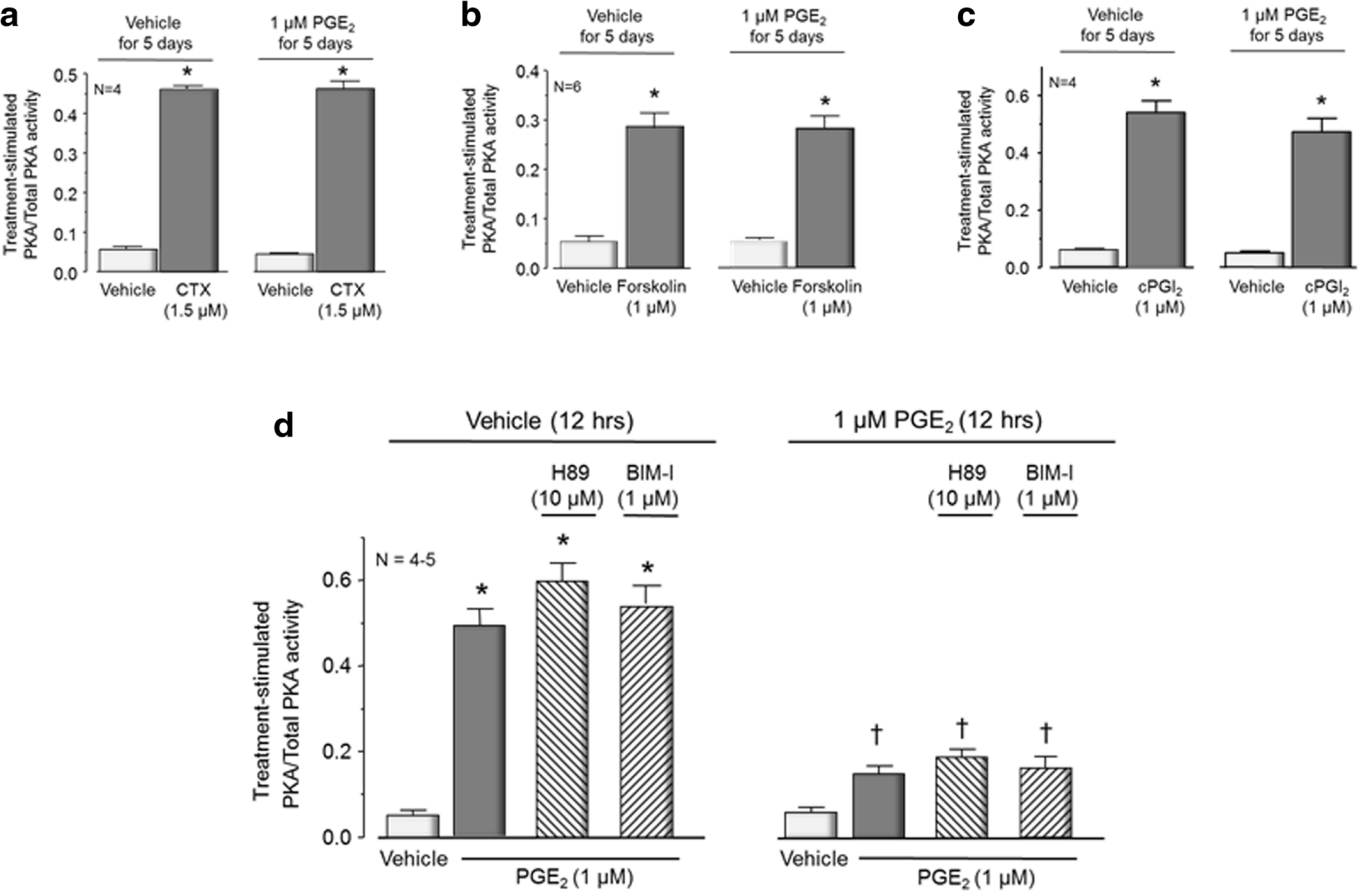
Five-day exposure of sensory neuronal cultures to PGE_2_ does not produce heterologous desensitization. Each column represents the mean ± SEM of the treatment-stimulated PKA activity normalized to total PKA activity in cultures exposed to vehicle for 5 days or PGE_2_ (1 μM) for 5 days as indicated. **a** Cultures were washed and then re-exposed for 10 min to vehicle (*open columns*) or CTX (1.5 μg/ml) overnight (*shaded columns*). **b** Cultures were washed and then re-exposed for 10 min to vehicle (*open columns*) or 1 μM forskolin for 20 min (*shaded columns*). **c** Cultures were washed and then re-exposed for 10 min to vehicle (*open columns*) or 1 μM cPGI_2_ (*shaded columns*) for 20 min. An *asterisk* indicates statistically significant difference from vehicle using one-way ANOVA followed by Bonferroni’s post hoc test. **d** The *left panel* represents PKA activity from cells exposed to vehicle for 12 h in the absence or presence of 10 μM H-89 or 1 μM BIM-I as indicated, while the *right panel* represents PKA activity from cells exposed to PGE_2_ (1 μM) for 12 h in the absence or presence of 10 μM H-89 or 1 μM BIM-I as indicated. After washing, the cells were re-exposed to vehicle (*open columns*) or 1 μM PGE_2_ (*shaded and hatched columns*) for 10 min. *Asterisks* indicate statistically significant differences in cells acutely exposed to vehicle versus cells exposed to PGE_2_. *Crosses* represent statistically significant differences in cells preexposed for 12 h to vehicle versus those exposed for 12 h to PGE_2_. Statistical analysis was performed using one-way ANOVA followed by Bonferroni’s post hoc test

Classical GPCR desensitization is mediated by receptor uncoupling from the cognate heterotrimeric G-protein and the downstream signaling pathway [13, 38]. Consequently, in sensory neurons exposed to PGE_2_ for 5 days, it is possible that the EP receptors are no longer coupled to the G_αs_/adenylyl cyclase/PKA pathway. If receptor uncoupling mediates the loss of PGE_2_-induced activation of PKA, then bypassing the receptor by directly activating G_αs_ or adenylyl cyclases should increase PKA activity even after long-term exposure to PGE_2_. To test this, we examined the effects of CTX or forskolin on PKA activity after long-term exposure of sensory neuronal cultures to PGE_2_. When neuronal cultures treated with vehicle for 5 days were exposed to 1.5 μg/ml CTX overnight, the PKA activity increased from 0.06 ± 0.007 (vehicle) to 0.46 ± 0.01 (Fig. 7a). In a similar manner, when cultures were exposed to PGE_2_ for 5 days, CTX increased PKA activity from 0.05 ± 0.003 (vehicle) to 0.46 ± 0.02 (Fig. 7a). When neuronal cultures treated with vehicle for 5 days were exposed to 1 μM forskolin for 20 min, the activator of adenylyl cyclases significantly increased PKA activity from 0.06 ± 0.01 to 0.28 ± 0.04 (Fig. 7b). In cultures treated with 1 μM PGE_2_ for 5 days, exposure to forskolin increased PKA activity from 0.05 ± 0.01 to 0.27 ± 0.04 (Fig. 7b). Thus, the downregulation of PGE_2_-activated PKA appears to result from the uncoupling between PGE_2_ and the PKA signaling pathway at the receptor level.

To determine whether the desensitization of the PGE_2_-induced PKA activation is heterologous with PGI_2_, we treated sensory neuronal cultures with vehicle or 1 μM PGE_2_ for 5 days and examined PKA activity after acute exposure to the stable analog of prostacyclin, cPGI_2_ [39]. We chose to examine this eicosanoid since it increases cAMP levels and sensitizes sensory neurons through activation of another GPCR, the IP receptor [7, 40]. In neuronal cultures treated with vehicle for 5 days, a 10-min exposure to 1 μM cPGI_2_ significantly increased PKA activity from 0.06 ± 0.004 to 0.544 ± 0.04. In an analogous manner, cPGI_2_ increased the PKA activity from 0.05 ± 0.002 to 0.48 ± 0.05 in neuronal cultures exposed to 1 μM PGE_2_ for 5 days (Fig. 7c). These data support the notion that the desensitization observed to PGE_2_-induced activation of PKA after long-term administration of the prostanoid is homologous.

Previous studies have shown that PKA can phosphorylate the β-adrenergic receptor and this can result in desensitization [41]. In an analogous manner, activation of PKC is associated with desensitization of IP receptors [42] and thromboxane receptors [43]. Consequently, after long-term exposure to PGE_2_, activation of PKA and/or PKC might result in phosphorylation and uncoupling of the EP receptors from their cognate G-proteins. To examine this, we treated sensory neurons in culture for 12 h with 1 μM PGE_2_ in the absence and presence of 10 μM H-89 or 1 μM BIM-I to block PKA or PKC activities, respectively, and then examined the effects of an acute challenge with PGE_2_. When neuronal cultures were treated for 12 h with vehicle in the absence or presence of H-89 or BIM-I, exposing the cultures to 1 μM PGE_2_ for 10 min caused a significant (~10-fold) increase in PKA activity compared to cells not exposed to the prostanoid (Fig. 7d). In contrast, in cultures exposed to 1 μM PGE_2_ for 12 h in the absence or presence of H-89 or BIM-I, re-exposure to the eicosanoid did not significantly increase PKA activity above basal levels (Fig. 7d). In cultures treated with PGE_2_ for 12 h and re-exposed to vehicle, the PKA activity was 0.06 ± 0.01, whereas with re-exposure to PGE_2_, the activity was 0.14 ± 0.02. In cultures treated with PGE_2_ and H-89 or PGE_2_ and BIM-I for 12 h, the PKA activity was 0.19 ± 0.02 or 0.16 ± 0.02 after re-exposure to the eicosanoid, respectively (Fig. 7d). These findings suggest that desensitization of the PGE_2_-induced activation of PKA after long-term exposure to the prostanoid is not mediated by PKA or PKC-induced phosphorylation of EP receptors.

### Acute PGE_2_-induced sensitization and persistent sensitization after long-term exposure to the eicosanoid are not mediated by activation of PI3 kinases

**Fig. 8:**
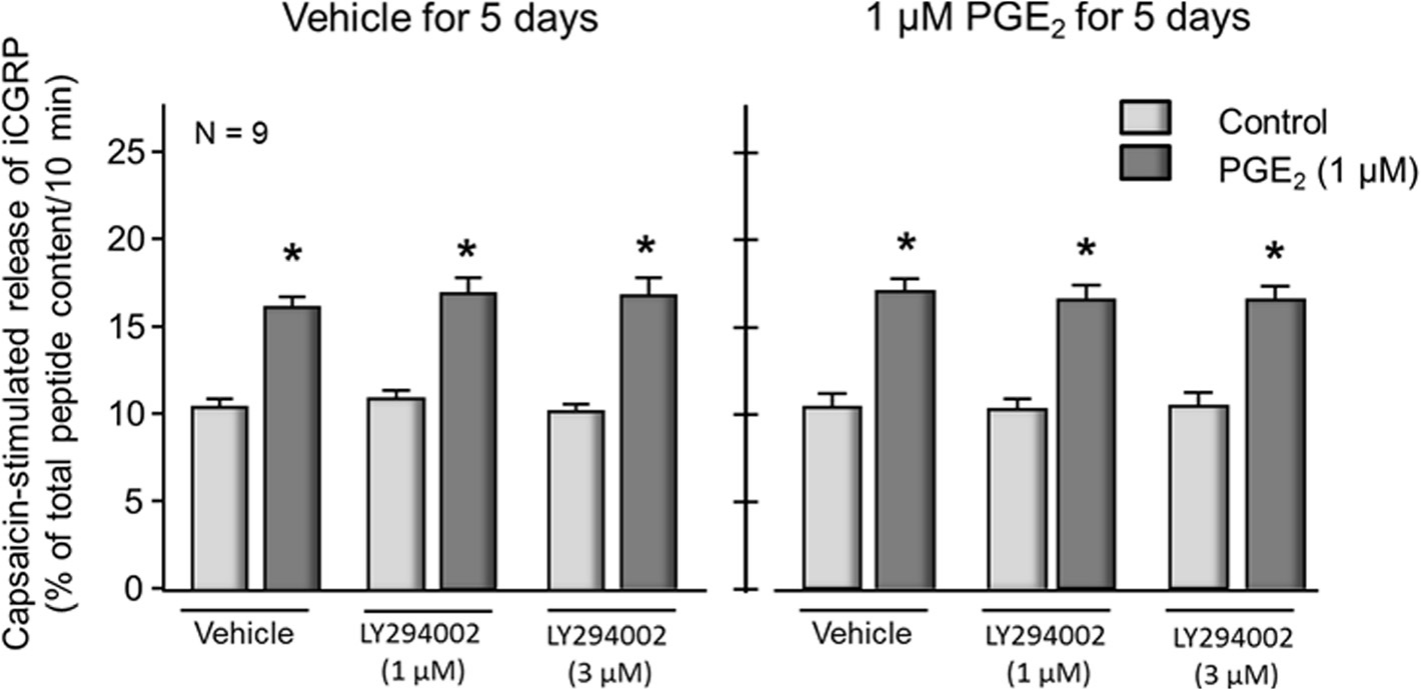
Acute PGE_2_-induced sensitization and persistent sensitization after long-term exposure to the eicosanoid are not mediated by activation of PI3 kinases. Each column represents the mean ± SEM of capsaicin-stimulated release of iCGRP as percent of total iCGRP content per well/10 min in cultures maintained in the absence of added NGF and preexposed to vehicle for 5 days (*left panel*) or to 1 μM PGE_2_ for 5 days (*right panel*). After long-term exposure, cultures were acutely exposed to vehicle (*lightly shaded columns*) or to 1 μM PGE_2_ (*dark-shaded columns*) for 20 min in the absence or presence of LY294002, as indicated. An *asterisk* indicates a statistically significant difference between capsaicin-stimulated release of iCGRP after vehicle versus after PGE_2_ using one-way ANOVA followed by Bonferroni’s post hoc test

The data presented above show that both acute and persistent sensitization of sensory neurons by PGE_2_ are mediated by activation of the same EP receptor subtypes but that sensitization after chronic exposure to PGE_2_ is not dependent on activation of PKA. Since previous work has shown that binding of PGE_2_ to EP4 receptors activates phosphoinositide 3-kinase (PI3K) signaling under different conditions [44] and that inhibiting PI3 kinases attenuates inflammatory pain behaviors [45, 46], we examined whether a pan inhibitor of PI3 kinases would attenuate acute or persistent PGE_2_-induced sensitization. In sensory neuronal cultures treated with vehicle for 5 days, exposing neuronal cultures to 1 or 3 μM LY294002 prior to and throughout exposure to capsaicin did not attenuate the ability of 1 μM PGE_2_ to augment stimulated iCGRP release (Fig. 8). In a similar manner, when sensory neurons were treated with 1 μM PGE_2_ for 5 days, then re-exposed to PGE_2_ neither 1 nor 3 μM LY294002 significantly altered the eicosanoid-induced increase in capsaicin-stimulated release of iCGRP.

## Discussion

The results presented here demonstrate for the first time that long-term exposure of sensory neuronal cultures to PGE_2_ results in a downregulation in the ability of the eicosanoid to activate PKA. This downregulation occurs rapidly with a significant loss of PKA activation within 3 h of exposure to 1 μM of the agonist and a complete loss within 72 h. Furthermore, it is reversible since within 24 h after removal of PGE_2_ from the neuronal cultures, the ability of PGE_2_ to increase PKA activity is fully restored. Long-term exposure of neuronal cultures to PGE_2_, however, does not diminish total PKA activity in the cells or the ability of CTX, which activates G_αs_ through ADP ribosylation, or forskolin, which activates adenylyl cyclases, to increase PKA activity. Exposing neuronal cultures to the selective EP4 receptor agonist L902688 also activates PKA, and a cross desensitization is observed with this agonist in neuronal cultures exposed to PGE_2_ for 5 days. This cross desensitization supports the notion that EP4 receptors are critical mediators of sensitization by PGE_2_. This observation is further substantiated by the finding that the EP4 receptor selective antagonist is capable of blocking sensitization caused by acute exposure to PGE_2_ and by re-exposure to PGE_2_ after long-term incubation with the eicosanoid.

The importance of PKA as an effector mediating acute sensitization of sensory neurons induced by PGE_2_ is well established. Increasing levels of cAMP in sensory neurons or exposure to cAMP analogs mimics the sensitizing actions of PGE_2_ in that the second messenger augments transmitter release from sensory neurons [7], increases the number of action potentials generated by various stimuli [47], sensitizes small unmyelinated sensory fibers to heat [48], increases TRPV1 channel activity [12], increases sodium current in sensory neurons [10, 11], and reduces potassium currents [49]. Inhibitors of PKA block hyperalgesia induced by PGE_2_ [50] and attenuate the acute sensitizing actions of PGE_2_ on sensory neurons [11, 12, 51, 52]. Although PKA is a critical effector of sensitization in sensory neurons after acute exposure to prostaglandins, it does not appear to be a major effector of persistent sensitization. Exposing the sensory neurons in culture to 1 μM PGE_2_ for 5 days does not alter the ability of the prostanoid to augment the capsaicin-stimulated release of the neuropeptide, CGRP from the neurons. With acute exposure to PGE_2_, the augmentation of transmitter release is blocked by pretreatment with the PKA inhibitor H-89. This compound has an IC_50_ for inhibition of PKA in the nanomolar range [53], and at the concentration we used, H-89 completely inhibits PKA activation in our cultures. Unlike the acute sensitizing actions of PGE_2_, however, in neurons pretreated with PGE_2_ for 5 days, H-89 does not block the sensitizing effects of PGE_2_. These data provide a mechanism to account for the observations in animal models that PGE_2_-induced sensitization does not downregulate with chronic exposure [54] and that after inflammation or chronic exposure to PGE_2_, the hyperalgesia produced by this prostanoid is not blocked by inhibitors of PKA [18, 20, 25].

Long-term exposure to PGE_2_ did not downregulate the ability of cPGI_2_ to activate PKA in sensory neurons, demonstrating that the PGE_2_-induced desensitization is homologous with respect to EP receptors. This finding is somewhat unexpected since both EP and IP receptors are expressed on sensory neurons and PGI_2_ produces hyperalgesia [55] and sensitization of sensory neurons through activation of the cAMP transduction cascade in a manner analogous to that of EP receptors [7, 40]. The lack of cross-desensitization, however, suggests that the PGE_2_-induced downregulation is not caused by activation of the second messenger-activated kinases, a mechanism which underlies heterologous desensitization [38, 56]. This is consistent with our observations that downregulation of PGE_2_-induced activation of PKA is not attenuated in neuronal cultures preexposed to 10 μM H-89 or to 1 μM BIM-I for 12 h during the exposure to PGE_2_. This concentration of H-89 is sufficient to totally inhibit PKA activity in the cultures, as well as the purified catalytic subunit of PKA in vitro (data not shown), and blocks the ability of acute PGE_2_ to sensitize the neurons. The concentration of BIM-I used in our experiments is sufficient to inhibit activity of classic and novel PKCs [57]. Therefore, it is logical to conclude that neither the two PKA isoforms PKA-I and PKA-II, which are inhibited by H-89 [58, 59], nor the classic or novel PKCs mediate the desensitization induced by long-term exposure to PGE_2_.

One interesting observation in the current work is that 10 μM isoproterenol only increases PKA activity modestly compared to 1 μM PGE_2_, cPGI_2_, forskolin, 1.5 μg/ml cholera toxin, or 300 nM L902688. Moreover, isoproterenol concentrations from 1 to 10 μM did not cause an appreciable difference in PKA activation, suggesting a lack of a concentration-response relationship. One possible explanation for the low levels of PKA activation by isoproterenol is that phosphodiesterase (PDE) activity could increase the breakdown of cAMP in the subcellular compartment in which PKA is localized [60, 61] since we did not include a PDE inhibitor in our assay buffer. Much evidence shows that scaffolding proteins, e.g., A-kinase anchor proteins (AKAPs), can maintain adenylyl cyclase, PKA, and PDE in close proximity, thus creating a highly localized, selective, and controlled signaling complex [62–64] which suggests that breakdown of cAMP could be a variable in controlling PKA activity. It seems unlikely, however, that this could account for the difference in PKA activation by isoproterenol versus PGE_2_ since previous reports indicated that activation of PKA by PGE_2_ is also subject to PDE suppression via degradation of cAMP [65, 66]. Moreover, PKA activity induced by either PGE_2_ (1 μM) or isoproterenol (10 μM) was assayed under the same experimental conditions. Thus, whether PKA-activation is subject to PDE suppression or not, we observed that isoproterenol is at least two orders of magnitude less potent than PGE_2_ in activation of PKA in isolated adult rat sensory neuronal cultures.

In the current experiments, we show that exposing the cultures to PGE_2_ for 5 days prevents a subsequent treatment with PGE_2_ from significantly increasing cAMP levels. This observation confirms previous work [24, 67, 68] and suggests that chronic exposure to PGE_2_ causes a downregulation of EP receptors or that the EP receptors are no longer effectively coupled to G_αs_. However, reduction of EP receptor expression cannot explain the loss of PGE_2_-induced cAMP production or PKA activation following long-term exposure to the eicosanoid, since it is evident from our data that neither EP receptor mRNA nor protein was significantly reduced after long-term exposure to PGE_2_. It is important to note that increases in cAMP that are sufficient to activate PKA are highly compartmentalized, through interaction with multiple AKAPs [69–71]. Consequently, the measure of total cAMP content in tissues may not reflect the functional effects of the second messenger.

We have previously shown that a 24 h exposure of sensory neuronal cultures to PGE_2_ significantly reduces the maximal receptor binding (Bmax) for the eicosanoid [24]. A similar decrease in Bmax of PGE_2_ occurs in the dorsal spinal cord after inflammation, and this effect is blocked by NSAIDs, suggesting it is secondary to prostaglandin production [24]. These data and our current finding that PKA activation is significantly downregulated after a 12-h exposure to PGE_2_ suggest that prolonged exposure to PGE_2_ results in downregulation of surface expression of EP receptors, presumably through internalization by the G-protein receptor kinase (GRK) and β-arrestin machinery [72, 73]. Despite the decrease in receptor binding, the ability of PGE_2_ to sensitize sensory neurons is not diminished and this is not likely due to a shift from EP receptors linked to G_αs_ to those linked to G_αq_ since a selective EP1 receptor antagonist does not block acute or persistent sensitization by PGE_2_. Furthermore, other investigators have shown that inflammation or exposure to PGE_2_ results in a modest increase in the expression of EP4 receptors on the plasma membrane in sensory neurons [34, 74, 75], although the reasons for the differences between our results and their findings remain to be determined. Consequently, it is unlikely that changes in receptor expression could account for a loss of the ability of PGE_2_ to activate PKA while maintaining the ability to sensitize the neurons. A more likely explanation is that after chronic PGE_2_, the signaling pathway mediating PGE_2_-induced sensitization switches from G_αs_ to other heterotrimeric G-proteins, such as G_αq/11_, or G_α12/13_ in a manner analogous to that observed with β-adrenergic receptors [76]. In the case of the EP4 receptors, studies in heterologous expression systems have shown that the receptor can couple to G_αs_ and G_αi/o_ under different conditions [77, 78]. Moreover, there is precedent to suggest that EP4 receptors may signal through G_βγ_ [79, 80]. In both cases, however, it is thought that PI3K relays the signal from either G_αi/o_ or G_βγ_ to downstream signaling pathways [44]. Nevertheless, LY294002 did not attenuate PGE_2_-induced sensitization after acute or long-term exposure to the eicosanoid, suggesting that PI3K does not contribute to PGE_2_-induced sensitization in sensory neurons.

It remains to be determined how PGE_2_ maintains its sensitization after long-term exposure to the eicosanoid. One possibility is that EP receptors, especially EP4, become phosphorylated on the C-terminus by GRKs [81] and that β-arrestins are recruited to EP4 receptors following exposure to PGE_2_ [82, 83]. β-arrestin-mediated signaling is well characterized and includes a wide array of signaling pathways [84], including, but not limited to, the MEK/ERK signaling pathway [85]. Thus, activation of as yet, undiscovered downstream signaling cascades might provide a means for sensitization to last after long-term exposure to PGE_2_. Further work is warranted to attempt to discover the downstream signaling mediating persistent sensitization since selective manipulation of such a pathway may prove useful in treating chronic inflammatory pain.

## Conclusions

Long-term exposure to PGE_2_ does not alter its ability to sensitize sensory neurons; however, the signaling pathway that mediates the sensitizing action of PGE_2_ is no longer dependent upon activation of PKA. Indeed, long-term exposure to PGE_2_ results in downregulation of the ability of PGE_2_ or the EP4 selective agonist, L902688, to activate PKA. This downregulation is reversible and homologous since it does not affect the ability of PGI_2_ to activate PKA. PGE_2_-induced sensitization after long-term exposure is largely mediated by EP4 receptor and is independent of both PKA and PI3K signaling pathways.

## Abbreviations

cAMP, 3′,5′-cyclic adenosine monophosphate; cPGI_2_, carbaprostacyclin; CTX, cholera toxin; GPCRs, G-protein-coupled receptors; iCGRP, immunoreactive calcitonin gene-related peptide; MPL, 1-methyl-2-pyrrolidinone; NGF, nerve growth factor; PDE, phosphodiesterase; PGE_2_, prostaglandin E_2_; PGI_2_, prostaglandin I_2_; PI3K, phosphoinositide 3-kinase; PKA, protein kinase A; PKC, protein kinase C; PKI 5-24, PKA pseudosubstrate inhibitor fragment 5-24
